# Effect of adipose-derived mesenchymal stem cells on the viability of the transverse rectus abdominis myocutaneous flap in rats

**DOI:** 10.1016/j.clinsp.2025.100590

**Published:** 2025-02-04

**Authors:** André Luiz Pires de Freitas, Sang Won Han, Priscila Keiko Matsumoto Martin, Lydia Masako Ferreira

**Affiliations:** a Division of Plastic Surgery, Universidade Federal de São Paulo (UNIFESP), São Paulo, SP, Brazil; b MSc Interdisciplinary Center for Gene Therapy (CINTERGEN), Universidade Federal de São Paulo (UNIFESP), São Paulo, SP, Brazil; c Department of Experimental Research, Hospital Israelita Albert Einstein, São Paulo, SP, Brazil

**Keywords:** Mesenchymal stem cell transplantation, Necrosis, Myocutaneous flap, Rectus abdominis, Rats

## Abstract

**Introduction:**

The Transverse Rectus Abdominis Myocutaneous (TRAM) flap is used for breast reconstruction, but involves the risk of necrosis. Adipose tissue-derived mesenchymal Stem Cells (ADSCs) can be used to stimulate neovascularization and reduce the risk of TRAM flap necrosis.

**Aim:**

Determine the effect of ADSCs on TRAM flap viability in rats.

**Methods:**

Twenty-four Wistar-EPM rats were distributed into three groups (*n* = 8). A right caudal pedicled TRAM flap was performed in all the animals and was the only procedure performed in Group TRAM. The additional procedures of intradermal injection of α-MEM culture medium and intradermal injection of α-MEM containing ADSCs labeled with a fluorescent marker were performed in Groups α-MEM and α-MEM-SC, respectively. The percentage of flap necrosis was determined, and the level of neovascularization and distribution of stem cells in the TRAM flap was assessed using immunohistochemical analysis and fluorescence microscopy, respectively.

**Results:**

The percentage of necrosis observed in Group α-MEM-SC was lower than that observed in Groups TRAM and α-MEM, namely 23.36 % vs. 50.42 % and 53.57 %, respectively (*p* < 0.05). In Zone IV of the flap, the number of vessels was greater in Group α-MEM-SC than in the other groups (*p* < 0.05). Multiple stem cells were observed in the four zones of the flap in Group α-MEM-SC. No stem cells were observed in Groups TRAM or α-MEM.

**Conclusion:**

ADSCs increased TRAM flap viability and the number of vessels in Zone IV of the flap in rats.

## Highlights

• ADSCs increased the viability (significantly less necrosis) of a TRAM flap in rats.
• Vessel formation in the ischemic segment of the flap was also enhanced by the ADSCs.
• Fluorescence microscopy indicated no differentiation of ADSCs into endothelial cells.

## Introduction

The Transverse Rectus Abdominis Myocutaneous (TRAM) flap is an option used for breast reconstruction.^1^, ^2^, ^3^ Its main advantage is the use of autogenous tissue, which gives the reconstructed breast a natural appearance.^2^^,^^3^ However, TRAM vascularization may be insufficient, and lead to complications, such as skin and adipose tissue necrosis.^2^, ^3^, ^4^ Increased vascularization, leading to increased TRAM viability, is particularly important to promote when other risk factors are involved, such as systemic arterial hypertension, diabetes mellitus, obesity, vascular disease, previous radiotherapy, smoking, and previous abdominal surgery.^4^^,^^5^

An alternative option for promoting increased vascularization is the flap delay procedure, whereby the TRAM flap is raised and transferred in two separate operative stages. The flap is submitted to partial ischemia in the first stage, and then transferred to its final position in a second surgical session, after observing a period of time which can vary from days to weeks.^6^ This technique has been reported to induce vascular reorganization, sympathectomy, angiogenesis, and the incorporation of new angiosomes, enhancing the vascularization of the flap.^7^^,^^8^ On the Other hand, it has the disadvantage of requiring a second, additional surgical procedure.

Advances in vascular biology have suggested some therapeutic techniques to increase the viability of ischemic tissues. Previous studies have investigated the possibility of inducing angiogenesis in non-ischemic tissues to increase vascularization, thus reducing postoperative ischemic complications.^9^^,^^10^ Three decades ago, a group of polypeptides with angiogenic action were identified, thus raising the possibility of new therapeutic perspectives. A previous study investigated an increase in TRAM flap viability in rats through gene therapy involving the Vascular Endothelial Growth Factor (VEGF), and observed less ischemia in the treated group.^11^ However, the effect of exogenous growth factor therapies may be limited owing to the reduced number of endothelial cells they produce. An alternative strategy would be to increase the number of endothelial cells to increase the formation of new blood vessels.^12^^,^^13^

It has already been demonstrated that Bone Marrow Mesenchymal Stem Cells (BMSCs) have the potential to differentiate into various cell types, including endothelial cells, thus representing an alternative to using endothelial progenitor cells in cell therapy procedures.^14^^,^^15^ Similarly, Adipose tissue-derived mesenchymal Stem Cells (ADSCs) can also transdifferentiate into endothelial progenitor cells, endothelial cells, and new blood vessels. The aim of this study was to evaluate the potential of a cell therapy technique based on using ADSCs to promote localized and long-lasting vascularization of TRAM flaps in rats, by assessing the viability of the flap, and the distribution of stem cells within it.

## Methods

This study was approved by the Research Ethics Committee of the Federal University of São Paulo (UNIFESP; opinion n° 0148/12). The animal experiments reported herein were conducted in full compliance with the Animal Research: Reporting of In Vivo Experiments (ARRIVE) guidelines, and with the National Research Council's Guide for the Care and Use of Laboratory Animals. Twenty-four male Wistar-EPM rats weighing between 350 g and 400 g were anesthetized by intraperitoneal injection of 25 mg/kg of tiletamine hydrochloride and zolazepam hydrochloride, immobilized on a surgical board in dorsal decubitus, and subjected to hair removal in the ventral region using an electric razor (Oster; McMinnville, TN, USA). No antibiotic prophylaxis was performed.

The animals were randomly distributed into three groups of eight animals each using an urn randomization program (UConn School of Medicine, Department of Public Health Sciences, Farmington, CT, USA). In Group TRAM, the animals underwent the TRAM flap procedure and intradermal injection of saline solution. In Group α-MEM, the animals underwent the TRAM flap procedure and intradermal injection of Eagle's minimum essential culture medium with alpha Modification (α-MEM). In Group α-MEM-SC, the animals underwent the TRAM flap procedure and intradermal injection of ADSCs labeled with a fluorescent marker in α-MEM.

### Obtaining adipose tissue

**Fig. 1:**
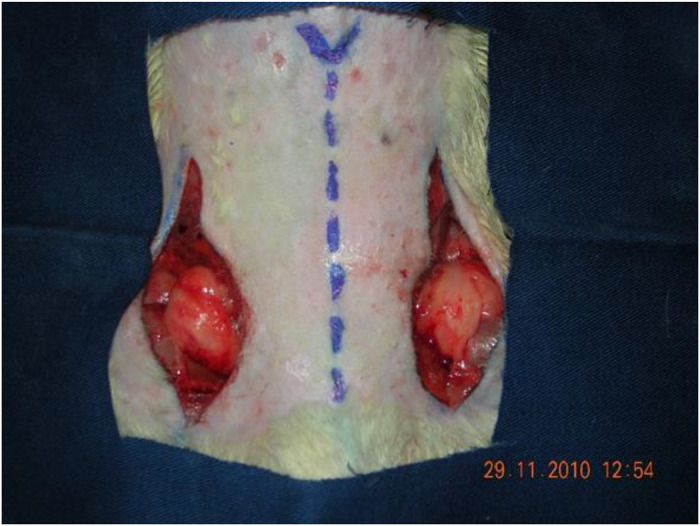
Surgical procedure for obtaining the adipose tissue used in the study. An incision was made along the entire inguinal fold of the rat, and then extended 2 cm superiorly along the anterior axillary line, at a distance of 3 cm from the midline.

The adipose tissue used in the study was removed from the bilateral inguinal region of the rat. An incision was made along the entire inguinal fold of the animal, extended 2 cm superiorly at the end along the anterior axillary line, and kept at a distance of 3 cm from the midline^16^ (Fig. 1).

### ADSC culture and identification

The excised adipose tissue was minced into pieces smaller than 2 mm using scissors and then incubated in a test tube with a 1 mg/mL type I collagenase solution for 3 h at 37 °C under agitation.^17^ The tube was subsequently centrifuged at 400 g for 10 min, and the supernatant was discarded. The cells were resuspended in Alpha MEM medium (Gibco, San Diego, CA, USA) supplemented with 200 mM L-glutamine (Gibco), 10,000 units/mL penicillin (Gibco), 10,000 units/mL streptomycin (Gibco), and 10 % Fetal Bovine Serum (FBS; Gibco). Subsequently, cell counting was performed using a Neubauer chamber with Trypan blue. After counting, the cells were incubated at a concentration of 1 × 10^6^ cells/cm^2^ and maintained at 37 °C in a 5 % CO_2_ atmosphere. The adherent cells remained in the same culture medium and under the same environmental conditions.

**Fig. 2:**
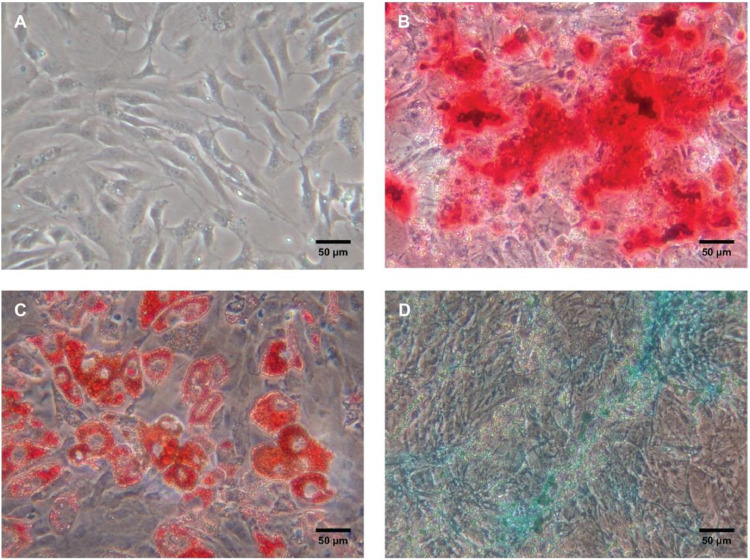
Differentiation of adipose tissue-derived mesenchymal stem cells. (A) Undifferentiated cells. (B) Osteogenic differentiation. (C) Adipogenic differentiation. (D) Chondrogenic differentiation.

The cells were characterized for the presence of surface markers, according to the manual of minimum criteria for characterizing mesenchymal stem cells.^18^ Cells from the third passage were analyzed by flow cytometry with antibodies positive for CD105, CD73, and CD90, and negative for CD45 and CD34. The cells were also evaluated with respect to both their proliferation as adherent cells, and their ability to differentiate into osteoblasts, adipocytes, and chondroblasts in vitro^18^ (Fig. 2).

### Labeling and preparation of stem cells

**Fig. 3:**
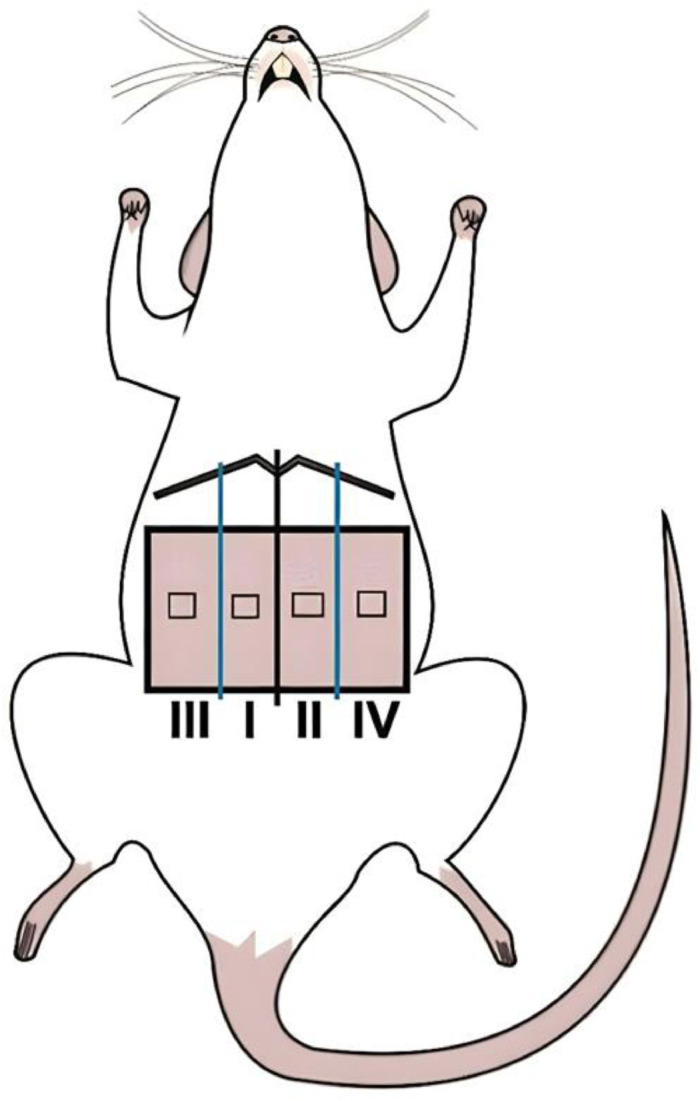
The four zones of the TRAM flap, showing the central biopsy sites.

The cells were treated with the Dil (1,1-dioctadecyl-3,3,3-,3-tetramethylindocarbocyanine) fluorescent marker to enable assessment of their location and distribution within the flap, by using fluorescence microscopy. The marker was added to the culture medium of adhered cells at a ratio of 1:1000, twelve hours before the surgical procedure. Only cultures containing stem cells isolated from the third passage were used in the in vivo study. The cells were resuspended in α-MEM medium at a concentration of 5 × 10^5^ cells per 0.1 mL, and then injected into the central points of Zones I through IV of the flap (Fig. 3), totaling 0.4 mL or 2 × 10^6^ cells per animal. The cells were injected intraoperatively during the TRAM flap elevation procedure.

### Animal model for an unipedicled TRAM flap

**Fig. 4:**
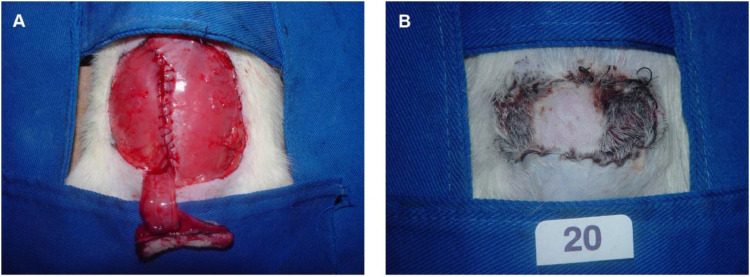
(A) A right inferiorly based pedicled TRAM flap. (B) The flap with extensive necrosis 7 days after the procedure.

The TRAM flap measured 3 × 5 cm and was positioned 1 cm caudally to the xiphoid appendix. The skin was incised, and detachment began on the contralateral side (left) of the pedicle and then progressed up to the linea alba. On the ipsilateral side (right) of the pedicle, the skin was dissected up to the lateral margin of the rectus abdominis muscle. A longitudinal incision was made along the aponeurosis of the linea alba and followed in the lateral margin of the muscle. Flap elevation was completed by severing the cranial muscular pedicle. The flap remained pedicled only to the right rectus abdominis muscle, and its irrigation was provided by the right deep caudal epigastric vessels^19^^,^^20^ (Fig. 4).

### Assessment of the necrosis area

The area of flap necrosis was assessed seven days after the procedure in all the groups.^21^ The flaps were photographed using a digital camera (Alpha-55, 35/70 mm; Sony, Tokyo, Japan), and the images obtained were analyzed using Image Tool software (University of Texas Health Science Center, San Antonio, TX, USA) to determine the percentages of flap necrosis.^11^

### Microscopic and immunohistochemical assessment

The flaps with their pedicles were sectioned to evaluate the possibility of thrombosis. A fragment was removed from the central area of each of the previously established zones of the TRAM^11^ (Fig. 3). All the tissues were evaluated for neovascularization by optical microscopy, using Hematoxylin & Eosin staining, and by immunohistochemical analysis, using the human heart factor 35 (HHF 35) marker. The number of vessels was expressed as vessels per field (400 ×; field area of 0.13 mm^2^). The distribution of mesenchymal stem cells in the flap was indicated by the presence of the Dil dye, and evaluated by fluorescence microscopy.

### Statistical analysis

The Shapiro-Wilk and Levene tests were used to check whether the data met the assumptions of normality and homoscedasticity, respectively. Data regarding the percentage of necrosis and the number of vessels (in the four zones and in the total flap) were evaluated by fixed-factor analysis of variance (ANOVA) and the Kruskal-Wallis test, respectively. Tukey's multiple comparison test was used to complement the ANOVA, as needed. The level of significance used was 5 %.

## Results

### Flap viability

The mean percentage of flap necrosis was 50.43 ± 23.35 %, 53.57 ± 28.40 %, and 23.36 ± 19.38 % in Groups TRAM, α-MEM, and α-MEM-SC, respectively. The percentage of flap necrosis was significantly lower in Group α-MEM-CT than in Groups TRAM and α-MEM (*p* = 0.012). There were no significant differences between Groups TRAM and α-MEM (*p* = 0.999; Table 1).

**Table 1:** Comparison of the study groups regarding the percentage of flap necrosis.

**Groups compared**		**Comparison outcome**	**p-value**
α-MEM-SC	TRAM	α-MEM-SC < TRAM	**0.012**
α-MEM-SC	α-MEM	α-MEM-SC < α-MEM	**0.012**
TRAM	α-MEM	TRAM = α-MEM	0.999

Fixed-factor analysis of variance (ANOVA) complemented by Tukey's multiple comparison test (*p* < 0.05).

### Immunohistochemical analysis of vessels

**Fig. 5:**
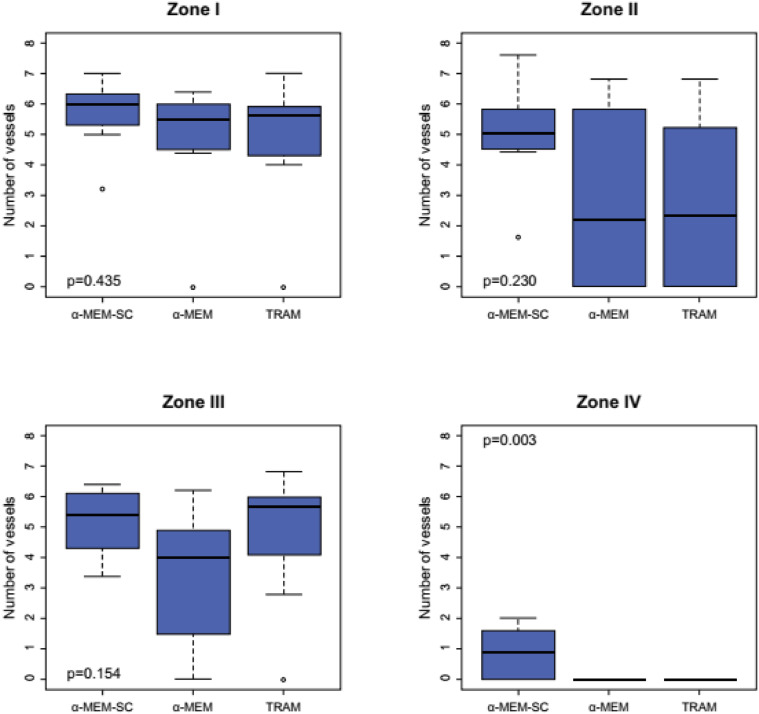
Boxplots of the number of blood vessels in Zones I through IV of the flap in Groups α-MEM-SC, α-MEM, and TRAM.

The vessels stained with HHF had an orange-brown color. The mean total number of vessels per field was 3.09 ± 1.37, 2.76 ± 1.50, and 4.18 ± 0.93 in Groups TRAM, α-MEM, and α- MEM-SC, respectively. There were no significant differences among the groups regarding the mean total number of vessels counted on the flap (*p* = 0.095). Fig. 5 contains boxplot graphs showing the behavior of this variable in the study groups according to flap zone. In Zone IV, the mean number of vessels was significantly higher in Group α-MEM-SC than in Group TRAM (*p* = 0.001) and Group α-MEM (*p* = 0.001). There was no significant difference among the groups in the other zones.

### Location and distribution of stem cells within the TRAM flap

**Fig. 6:**
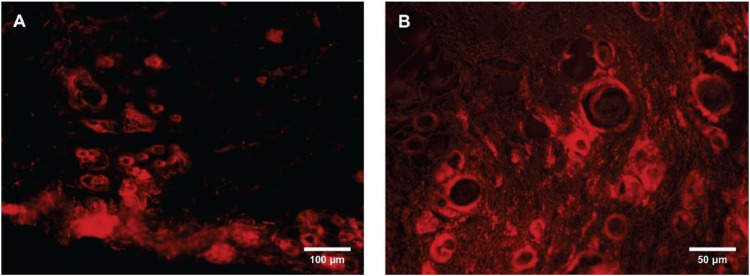
Dil-positive cells observed under fluorescence microscopy. (A) Zone IV of the TRAM flap, shows Dil-positive stem cells located around the blood vessels. (B) Zone III of the flap.

Multiple Dil-positive stem cells were observed in all the slides and in all four zones of the flap (I, II, III, and IV) in the α-MEM-SC animals. Animals from the TRAM and α-MEM groups had no fluorescently labeled stem cells, hence ruling out the need for any statistical comparison among the groups. Stem cells (Dil positive) were found near and around blood vessels in 10 of the 32 slides from Group α-MEM-SC; in the majority of these slides (8 of 10), the cells were located in Zones III and IV (Fig. 6). No Dil-positive stem cells were observed in the blood vessel walls.

## Discussion

The TRAM flap is considered an important option for breast reconstruction with autologous tissue^1^, ^2^, ^3^^,^^22^; however, its use involves the risk of complications related to changes in flap vascularization.^3^^,^^4^ An alternative option for increasing flap vascularization is surgical delay, which involves an initial procedure where the flap is submitted to partial ischemia, and then a second procedure where the flap is transferred to its final position.^6^ The goal of this technique is to stimulate vascular reorganization, sympathectomy, angiogenesis, and the incorporation of new angiosomes, thereby enhancing the vascularization of the flap.^7^^,^^8^ Its disadvantages, however, are related to the introduction of an additional surgical session.

Several authors have investigated alternatives to the surgical delay procedure, e.g. chemical flap delay methods. Clugston et al.^23^ observed an increase in TRAM flap viability in rats after using allopurinol. Karacaoglu et al.^24^ applied epinephrine-loaded microspheres to the TRAM flap and observed similar viability levels to those promoted by surgical delay. In the present study, a novel procedure designed to increase vascularization of the flap by using mesenchymal stem cells intraoperatively was evaluated. The study investigated both the increase in the number of vessels and the potential transdifferentiation of ADSCs into endothelial cells. In a previous study utilizing the same model combined with VEGF gene therapy, the authors observed a significant enhancement in vascularization. However, this outcome was solely attributed to an increase in the number of capillaries.^11^

Stem cell-based therapies continue to evolve and play an increasingly important role in meeting the growing need for tissue repair and replacement. The prospect of using mesenchymal stem cells to increase vascularization of the TRAM flap led to the present study.

Zheng et al.^25^ demonstrated the neovascularization potential of BMSCs; however, the pro-angiogenic effect of ADSCs is based on a different mechanism from the pro-angiogenic effect of BMSCs, and involves neoangiogenesis, vasculogenesis, and the release of growth factors and cytokines, mainly VEGF, Fibroblast Growth Factor (FGF), Hypoxia-Inducible Factor-1α (HIF-1α), Transforming Growth Factor Beta (TGF-β), Stromal cell-Derived Factor-1α (SDF-1α), and Chemokine Receptor type 4 (CXCR4).^26^ These characteristics of ADSC, combined with both the use of minimally invasive techniques for their extraction and the ease involved in their isolation and culture, have favored the choice of this cell type for conducting experiments.^27^^,^^28^

A dose of 2 × 10^6^ cells was used for each animal in the present study. This dose was divided into equal parts (5 × 10^5^) for the four central points of the vascularization zones of the TRAM flap chosen for analysis, similar to a previous study on VEGF.^11^ Doses ranging between two hundred thousand and ten million cells can be found in the literature,^29^^,^^30^ but no study was found using intradermal cell therapy to increase the viability of the TRAM flap.

The allogeneic mesenchymal stem cells used came from three donor animals. The use of allogeneic cells was possible because of the immune privileged state of stem cells, a characteristic that renders these cells capable of immunomodulating inflammatory reactions and even the transplant rejection process, as observed in previous studies.^15^^,^^25^ Other studies have demonstrated the effectiveness of human-derived xenogeneic cells in reducing flap necrosis.^30^, ^31^, ^32^ Literature reviews conducted by Li et al.^33^ and Avila et al.^34^ showed that the effectiveness of cells of human origin in reducing flap necrosis in animal models was equal to, or greater than, the effectiveness of cells of non-human origin.^33^^,^^34^

The Dil fluorescent marker was used to label the stem cells and observe their distribution in the flap and/or their integration into the wall of blood vessels. The same strategy was used by Lu et al.^21^ and Gao et al.,^30^ found labeled cells arranged around blood vessels and cells within the wall of blood vessels, suggesting transdifferentiation of stem cells into endothelial cells. Karathanasis et al.^35^ used the Green Fluorescent Protein (GFP) as a marker and antibody for the Von Willebrand factor, and identified cells in the wall of blood vessels; however, these authors did not report on the immunophenotyping of the cells. This could mean that other cell types, such as pre-adipocytes, endothelial progenitor cells, and leukocytes were involved, in addition to the mesenchymal stem cells.^35^

The present study used the TRAM flap model. Similarly to other studies, it used the right caudal vascular pedicle, which is the non-dominant pedicle in rats, and best represents the TRAM procedure conducted in humans, which uses the upper pedicle—also non-dominant.^19^^,^^20^ The time chosen for the induced death of the animals and the beginning of the assessments was seven days, a time similar to that used in other studies with flaps in rats.^21^^,^^36^, ^37^, ^38^, ^39^, ^40^ This seven-day interval allowed analysis of the necrosis without risk of scar dehiscence or retraction. It also enabled the production of marker fluorescence under fluorescence microscopy, and ensured enough time for the differentiation of the mesenchymal stem cells into endothelial cells (vasculogenesis), as described in other studies.^21^^,^^30^^,^^35^

The mean percentage of TRAM flap necrosis was significantly lower in Group α-MEM- SC than in Groups α-MEM and TRAM, and the latter groups had similar mean percentages of necrosis. This result is similar to that obtained by Lu et al.,^21^ who, although working with a dorsal random flap, failed to observe any difference between the groups treated with saline solution or culture medium in terms of flap viability, thus showing that the culture medium did not affect this variable. The flap necrosis found mainly in the TRAM and α-MEM groups was probably caused by injury to the perforating vessels during flap detachment, leading to ischemia and necrosis, mainly of the extremities (Zones III and IV).

Although the necrosis assessment was performed seven days after the induced death of the animals ‒ a seemingly short time for differentiation of stem cells into endothelial cells and blood vessels to occur ‒ the flap in the animals from Group α-MEM-SC showed enhanced viability. Hasdemir et al.^37^ found similar results using the same number of stem cells and the same assessment time in a dorsal skin flap model. Schlosser et al.^29^ used an ischemic model and found a large angiogenic reaction on the seventh day after applying the stem cells.

There are several ways whereby ADSCs can promote increased flap viability, including transdifferentiation of ADSCs into endothelial cells and secretion of chemical mediators and growth factors.^26^^,^^41^ In the present study, flap viability was seemingly not ensured solely by an increase in the number of vessels. The paracrine effect of stem cells on an ischemic area, which is linked to several mechanisms, may be an additional factor involved in increased flap viability. Studies using an ischemia-reperfusion model observed a reduction in the corresponding lesion as a result of the action of growth factors, especially VEGF, FGF, TGF-β, and expression of IL-6.^32^^,^^42^ Gao et al.^30^ showed that the use of ADSCs can improve skin flap viability in diabetic mice via HIF-1α and VEGF expression. Yue et al.^41^ demonstrated that the preoperative transplantation of ADSCs, combined with hypoxic preconditioning of the flap, effectively improved ischemic flap viability in rats by neovascularization associated with the production and activation of HIF-1α, and with an increase in VEGF. Other studies have shown that mesenchymal stem cells increased flap viability by promoting angiogenesis, which is regulated by the microRNA-590–3p/VEGFA axis.^43^

There was no significant difference among Groups α-MEM-SC, TRAM, and α-MEM regarding the total number of blood vessels in the flap. Hasdemir et al.^37^ also found no significant differences among the study groups regarding the number of vessels, although they did observe that flaps treated with stem cells showed greater viability. A separate assessment of the number of vessels in the different flap zones revealed a significantly higher number of vessels for the α-MEM-SC group than for the TRAM and α-MEM groups in Zone IV. Freitas et al.^11^ also found a significantly greater number of vessels in Zone IV, highlighting the importance of treating this ischemic zone for overall flap viability. The animals in Groups TRAM and α-MEM had no vessels in Zone IV of the flap, resulting in necrosis in this area. Zone IV of the flap is considered an ischemic area in both human and animal models.

Fluorescence assessment of cells previously labeled with Dil made it possible to investigate the presence of stem cells after seven days, their distribution in the flap, and their incorporation into the vessel walls. Fluorescently labeled cells were found only in the α-MEM-SC group. Even though this result was expected, an assessment of the slides from the other groups was performed anyway to rule out the possibility of tissue autofluorescence. Other studies have shown increased vascularization and differentiation of stem cells into endothelial cells.^25^^,^^44^ Although both an increase in the vascularization of Zone IV of the flap and the presence of fluorescent cells close to the blood vessel walls were observed in the present study, Dil-positive cells were not found in blood vessel walls, thus questioning whether stem cell differentiation into endothelial cells actually took place. Similar results were found by Gao et al.,^30^ who reported an increased number of stem cells around blood vessels and few cells in vessel walls. Hasdemir et al.^37^ used the same transplant route, amount of cells, and evaluation time, and found GFP-labeled stem cells in the flap, but did not report integration of the cells into vessel walls. Schlosser et al.^29^ used BMSCs in an ischemic flap and observed an increase in viability, but failed to observe stem cell transdifferentiation into endothelial cells. On the fourth postoperative day, the cells were found gathered along the vascular walls, and, on the seventh day, a major angiogenic reaction began.^29^

In the present study, although there was no significantly greater number of blood vessels in Group α-MEM-SC compared to the control in Zones I, II, and III, there was an increase in flap viability. This increase in total viability may be a consequence of angiogenesis occurring in Zone IV and/or the paracrine action of stem cells in all areas of the flap. Previous studies found a significant increase in VEGF and HIF-1α in ischemic flaps and an increase in the expression of these two factors in dermal fibroblasts.^30^^,^^41^^,^^45^

Mesenchymal stem cells, or “medicinal signaling cells”, are home on sites of injury or disease and secrete bioactive factors that are immunomodulatory and trophic (regenerative), meaning that these cells produce therapeutic molecules *in situ* that are medicinal. The tissue-specific resident stem cells then construct the new tissue, stimulated by the bioactive factors secreted by the exogenously supplied MSCs.^46^

Further investigation is needed to address the questions raised in the present study. Future studies could investigate the correlation between TRAM flap viability and the use of endothelial progenitor cells, human stem cells, VEGF, HIF-1α, and pre-vascularized stem cells, similar to the study conducted by Zhou et al.,^47^ as well as the use of exosomes released by mesenchymal stem cells.^45^ The potential synergistic effect of combining surgical delay either with mesenchymal stem cells or with the stromal vascular fraction, or even with the mechanically isolated stromal vascular fraction (nanofat emulsification technique) containing additional adjuvant factors such as VEGF should also be explored as therapeutic alternatives. Furthermore, future investigations using different numbers of ADSCs and evaluation periods are recommended to explore how changes in these parameters could impact angiogenesis. These approaches could potentially result in an enhanced cost-benefit ratio for the TRAM flap procedure.

The observations presented herein were based on an experimental model, and cannot be immediately transferred to the clinical reality of breast reconstructions. The present study merely suggests the existence of important correlations and proposes opening new avenues of research for examining the issues related to postoperative complications.

## Conclusion

ADSCs increased the number of blood vessels observed in Zone IV of the TRAM flap in rats, and increased flap viability. No labeled stem cells were found in the walls of capillaries or other vessels.

## Funding

This research did not receive any specific grant from funding agencies in the public, commercial, or not-for-profit sectors.

## Declaration of competing interest

The authors have no financial interest or commercial association with any of the subject matter or products mentioned in this manuscript.
